# Characterization of a genetic mouse model of lung cancer: a promise to identify Non-Small Cell Lung Cancer therapeutic targets and biomarkers

**DOI:** 10.1186/1471-2164-15-S3-S1

**Published:** 2014-05-06

**Authors:** Federica Riccardo, Maddalena Arigoni, Genny Buson, Elisa Zago, Manuela Iezzi, Dario Livio Longo, Matteo Carrara, Alessandra Fiore, Simona Nuzzo, Silvio Bicciato, Patrizia Nanni, Lorena Landuzzi, Federica Cavallo, Raffaele Calogero, Elena Quaglino

**Affiliations:** 1 Molecular Biotechnology Center, University of Torino, 10126 Torino, Italy; 2 Department of Biotechnology, University of Verona, 37134 Verona, Italy; 3 Department of Oncology and Neurosciences, G. d'Annunzio University, 66100 Chieti, Italy; 4 Department of Chemistry IFM and Center for Molecular Imaging, University of Turin, 10126 Turin, Italy; 5 Center for Genome Research, Dept. of Life Sciences, University of Modena and Reggio Emilia, 41100 Modena, Italy; 6 Department of Medicina Specialistica, Diagnostica e Sperimentale, University of Bologna, 40126 Bologna, Italy; 7 Laboratory of Experimental Oncology, Rizzoli Orthopaedic Institute, 40136 Bologna Italy

## Abstract

**Background**

Non-small cell lung cancer (NSCLC) accounts for 81% of all cases of lung cancer and they are often fatal because 60% of the patients are diagnosed at an advanced stage. Besides the need for earlier diagnosis, there is a high need for additional effective therapies. In this work, we investigated the feasibility of a lung cancer progression mouse model, mimicking features of human aggressive NSCLC, as biological reservoir for potential therapeutic targets and biomarkers.

**Results**

We performed RNA-seq profiling on total RNA extracted from lungs of a 30 week-old K-ras^LA1^/p53^R172HΔg ^and wild type (WT) mice to detect fusion genes and gene/exon-level differential expression associated to the increase of tumor mass. Fusion events were not detected in K-ras^LA1^/p53^R172HΔg ^tumors. Differential expression at exon-level detected 33 genes with differential exon usage. Among them nine, i.e. those secreted or expressed on the plasma membrane, were used for a meta-analysis of more than 500 NSCLC RNA-seq transcriptomes. None of the genes showed a significant correlation between exon-level expression and disease prognosis. Differential expression at gene-level allowed the identification of 1513 genes with a significant increase in expression associated to tumor mass increase. 74 genes, i.e. those secreted or expressed on the plasma membrane, were used for a meta-analysis of two transcriptomics datasets of human NSCLC samples, encompassing more than 900 samples. SPP1 was the only molecule whose over-expression resulted statistically related to poor outcome regarding both survival and metastasis formation. Two other molecules showed over-expression associated to poor outcome due to metastasis formation: GM-CSF and ADORA3. GM-CSF is a secreted protein, and we confirmed its expression in the supernatant of a cell line derived by a K-ras^LA1^/p53^R172HΔg ^mouse tumor. ADORA3 is instead involved in the induction of p53-mediated apoptosis in lung cancer cell lines. Since in our model p53 is inactivated, ADORA3 does not negatively affect tumor growth but remains expressed on tumor cells. Thus, it could represent an interesting target for the development of antibody-targeted therapy on a subset of NSCLC, which are p53 null and ADORA3 positive.

**Conclusions**

Our study provided a complete transcription overview of the K-ras^LA1^/p53^R172HΔg ^mouse NSCLC model. This approach allowed the detection of ADORA3 as a potential target for antibody-based therapy in p53 mutated tumors.

## Background

Lung cancer is the most common cause of neoplasia-related death worldwide [1]. The vast majority of lung cancer cases (approximately 80%) are non-small cell lung cancers (NSCLC) and the remaining fraction is small cell lung cancers. Only a minority of NSCLC patients is suitable for radical treatment as curative care. Approximately two thirds of patients are diagnosed at an advanced stage, and of the remaining patients who undergo curative surgery, 30-50% have a recurrence with metastatic disease [2]. The 5-year relative survival rate among patients diagnosed with NSCLC is only 15%. Thus, the conventional treatments (i.e. surgery, radiotherapy and chemotherapy), have apparently reached a plateau of effectiveness in improving survival of advanced NSCLC patients [3]. Thus, the treatment of NSCLC is a major unmet need and new therapies focusing on the molecular mechanisms of lung tumorigenesis are urgently needed [4].

The discovery of new biomarkers for targeted therapies could greatly change the management and prognosis of many patients with NSCLC. Further, knowledge of the molecular pathways and mutational drivers of lung cancer will expand the use of targeted treatments. Hopefully, the identification of new therapeutic targets will provide personalized and precise treatments for lung cancer patients in the near future.

Indeed, considerable efforts were made to discover new molecular biomarkers associated to lung cancer, which could be used as early diagnostic markers or as new specific therapeutic targets to treat patients [5-7]. In our opinion, the identification of oncoantigens (i.e. tumor associated antigens that have a causal role in the promotion of tumor progression) [8,9] could provide new and more promising targets for personalized treatment in NSCLC.

In this study, we sought to identify new candidate biomarkers and/or potential oncoantigens involved in both initiation of lung cancer and/or its progression to an aggressive cancer phenotype. To this aim, we adapted to the lung cancer disease our consolidated pipeline for oncoantigen detection [8,10]. Thanks to the RNA-seq technology we also extended our pipeline to the detection of tumor specific transcript isoforms and fusion proteins [11]. Our pipeline requires the availability of an animal model for the cancer under study [8]. Thus, we used one of the models most closely simulating human metastatic lung cancer [12]. This model is based on the combination of a latent mutant K-ras allele at the endogenous locus (K-ras^LA1^), which is spontaneously activated in vivo [13], and a particular mutation generated at the endogenous p53 allele containing an arginine-to-histidine substitution at codon 172 (p53^R172HΔg^), corresponding to the hot spot mutation at human codon 175 [14-16]. This mouse model develops lung adenocarcinomas with a high incidence of metastases and gender differences in cancer-related death. The use of our pipeline in the framework of metastatic lung cancer model, combined with the power of RNA-seq technology, allowed the identification of ADORA3 as new putative target for antibody-based therapy in mutant p53 tumors.

## Results and discussion

### Characterization of lung tumors of K-ras^LA1^/p53^R172HΔg ^mice by non invasive MRI

**Figure 1:**
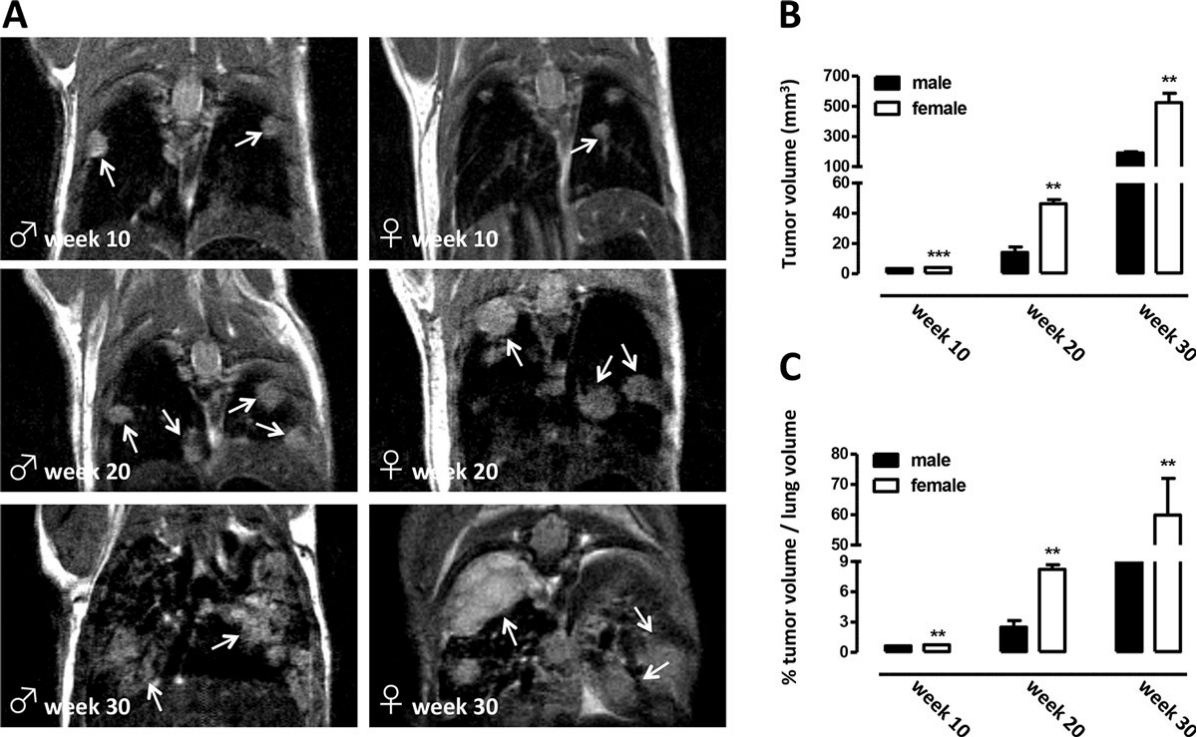
**Non-invasive imaging techniques (MRI) for small rodents**. A: T^2^weighted images of the lungs from 10, 20 and 30 weeks old K-ras^LA1^/p53^R172HΔ ^males (left panels) and females (right panels) mice. Tumors appear as white opaque hyper-intense regions (white arrows). B and C: Quantification of the tumor burden of both males (black bars) and females (white bars) mice at 10, 20 and 30 weeks of age. B: Tumor volume per animal was quantified by calculating the area of visible lung opacities present in each axial image sequence (usually 18-20 per mouse) and then multiplying the total sum of the areas by the distance between each MRI sequence. Data are shown as mean ± SEM of the areas occupied by the tumors in the lung of each mouse (** p = 0.005, *** p = 0.0001, Student' t test). C: Percentage of lung volume occupied by tumors; data are shown as mean ± SEM of each mouse (** p = 0.005, Student' t test).

A colony of K-ras^LA1^/p53^R172HΔg ^double transgenic mice has been generated in our laboratory, by crossing one p53^R172HΔg ^male with one K-ras^LA1 ^female, kindly provided us by Dr. Lozano (University of Texas, M.D. Anderson Cancer Center). These mice develop autochthonous lung adenocarcinomas with a high incidence of metastases and gender differences in cancer related death thus providing a realistic model of human metastatic lung cancer and an immunocompetent system for studying NSCLC and its prevention by novel agents [12]. By using non-invasive imaging techniques (MRI) for small rodents, a quantification of the number and the size of tumor lesions of K-ras^LA1^/p53^R172HΔg ^mice during time was performed. The progression of lung tumors was monitored at 10, 20 and 30 weeks of age. Tumor lesions resulted as white opaque hyper-intense regions already evident in 10 week-old K-ras^LA1^/p53 ^R172HΔg ^male and female mice (Figure 1A). The analysis of images collected at weeks 10, 20, and 30 weeks of age showed a significant increase in the total tumor volume in both K-ras^LA1^/p53 ^R172HΔg ^males and females during cancer progression (Figures 1B and 1C). Moreover, starting from the 10^th ^week of age, a significant increase in the number and size of lung lesions was observed between males and females, with females developing more lesions than males, as previously reported for survival [12]. These gender differences remain evident from early to advanced/late-stage of the disease (Figures 1B and 1C).

**Figure 2:**
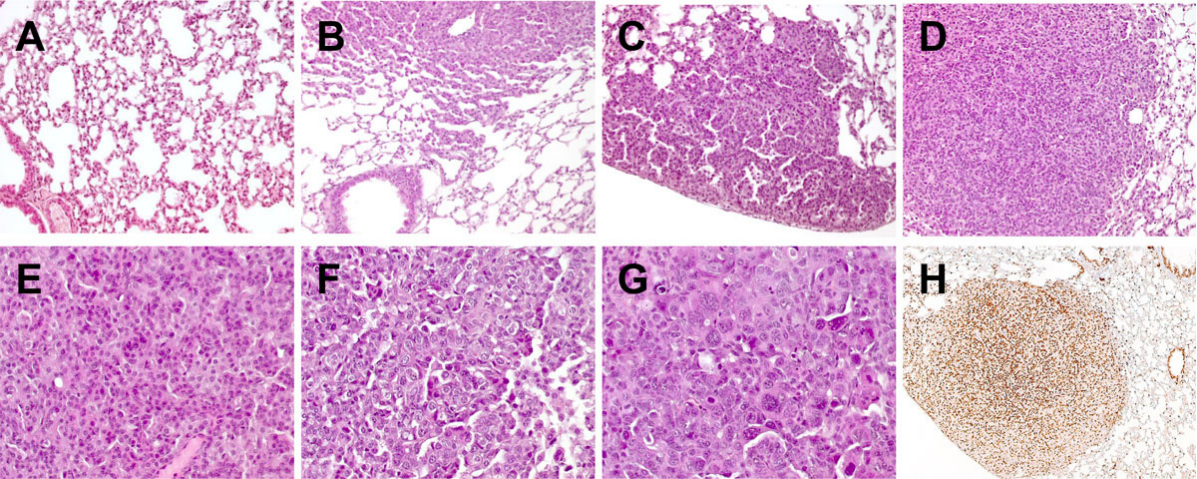
**Morphological characterization of lung tumors from K-ras^LA1^/p53^R172HΔg ^mice**. A-G: Hematoxylin-eosin evaluation of lung sections from a WT transgenic mouse (A), one representative 10- (B), 20- (C) and 30- (D-G) week-old K-ras^LA1^/p53^R172HΔg ^mice (A-D magnification ×200; E-G magnification ×400). A: normal lung tissue; B: initial lesions with aspects of lepidic growth; C: subpleural lesion with papillary and solid patterns; D: adenocarcinoma nodule with solid pattern of growth; E: tumor zone with a solid growth pattern composed of cohesive cell agglomerates in a nest-like configuration without acinar polarity; F: tumor zone with papillary growth. Papillae show fibrovascular cores lined by cells with large vesicular nuclei containing very prominent nucleoli; G: poorly differentiated tumor zone with highly polymorphic cells and cells with aberrant nuclei. H: Immunohistochemical staining for TTF-1 lung tumor lesions from one representative 30-week-old K-ras^LA1^/p53^R172HΔg ^mouse (magnification ×100).

Histological analysis of lung sections from normal (Figure 2A) and 10 week-old K-ras^LA1^/p53^R172HΔg^ male and female mice showed that white opacities revealed by the MRI analysis correspond to small foci of lung carcinoma growing with lepidic aspect (Figure 2B). These early lesions increase in number and dimensions and, at 20 weeks of age, become sub-pleural and intra-parenchymal tumors (Figure 2C and 2D, respectively), growing in masses with lepidic and solid growth aspects. Like in humans, in which the prevalence of adenocarcinomas of mixed subtypes led, in 2011, to a new WHO classification in which invasive adenocarcinomas are classified by predominant pattern and to the routinely definition of the percentage of histologic subtypes in clinical pathological reports, at 30 weeks of age (Figure 2E,2F,2G,2H), lung adenocarcinomas of K-ras^LA1^/p53^R172HΔg ^mice display, besides a predominance of zones with solid growth (Figure 2E), several types of differentiation, sometimes with prominent papillary growth pattern (Figure 2F), sometimes with less differentiated zones and aspects of large cell carcinoma (Figure 2G). Immunohistochemical analyses showed that these lesions are positive for TTF-1 (Thyroid Transcription Factor-1; Figure 2H), a typical marker of adenocarcinoma [17], and negative for p63, a marker of squamous tumors and for Synaptophysin, Chromogranin, and Neuron Specific Enolase (NSE; data not shown), markers of neuroendocrine tumors [18].

## Transcription profiling

### Microarray analysis

To estimate the importance of the gender effect on gene expression, we initially run a microarray experiment on lung tissues of 10, 20 and 30 week-old K-ras^LA1^/p53^R172HΔg ^mice, using Affymetrix exon 1.0 arrays. The comparison did not show any significant difference at the transcription level (not shown), suggesting that the differences in growth rate might be due to the endocrinological differences existing between male and female. Thus, we run a pair-end RNA-seq on two prototypical situations, WT and K-ras^LA1^/p53^R172HΔg ^mice (MT), to detect genes/transcripts associated to the increase of tumor mass that might represent potential targets for precision medicine applications [19].

### Fusion events detection

Direct sequencing of messenger RNA transcripts using the RNA-seq protocol [20] is rapidly becoming the standard method for detecting and quantifying expressed genes in a cell. One of the key features observed after cancer genomes analysis is a chromosomal abnormality. Genome rearrangements could result in aberrant gene fusions, and a number of them have been found to play important roles in carcinogenesis [21]. The discovery of novel gene fusions can lead to a better comprehension of cancer progression and development. Fusion events were detected in WT and MT samples using ChimeraScan [22]. Since fusion detection tools are error prone [23], we filtered the putative fusions, reported by ChimeraScan, retaining only common events between the MT and not reported in the WT replicates. The detected fusions (AK029407:Ank3, Gimap1:Gimap5, Pisd-ps2:Pisd-ps) were subsequently discarded since they were all either read through events or fusions between homologue genes. Thus it seems that fusion products are not prominent events in tumors developing due to the presence of constitutively active K-ras and inactive p53.

### Exon-level analysis

Exon level analysis was run using DEXSeq Bioconductor package [24] and provided 33 genes with differential exon expression between WT and MT groups (FDR < 10%). Among them six (ITGAD, COL17A1, DCSTAMP, PTPRN, PTPRM and Klrb1c) codify for proteins that were located on the plasma membrane and three (VWF, DMKN and TIMP3) for proteins secreted in the extracellular space. For 11 of the 33 detected genes, exon-level data for 509 tumors together with their clinical annotation were retrieved from the cancer genome atlas (http://cancergenome.nih.gov/). We scored the exons for their oncological power (see methods), which essentially represents the association between exon skipping/retention and poor outcome. Significant correlation between exon-level expression for the above-mentioned genes and poor prognosis could not be detected (not shown).

### Gene-level analysis

Gene-level analysis was run using DESeq Bioconductor package [25] and provided 1,513 genes with increased expression associated to tumor mass increment between WT and MT groups (FDR < 10%, |log_2_FC| > 1). We focused our analysis on 74 genes encoding for secreted and membrane bound proteins having a human ortholog (74). Thus, we run a meta-analysis on a set of public available transcriptomes of 989 NSCLC patients characterized by clinical outcome for survival and metastasis (see methods). The data set was divided in test and validation set, of 695 and 294 samples each, respectively. We scored the identified genes for their oncological power (CO score, see methods), which represents the association between up-modulation of a gene and poor clinical outcome.

**Figure 3:**
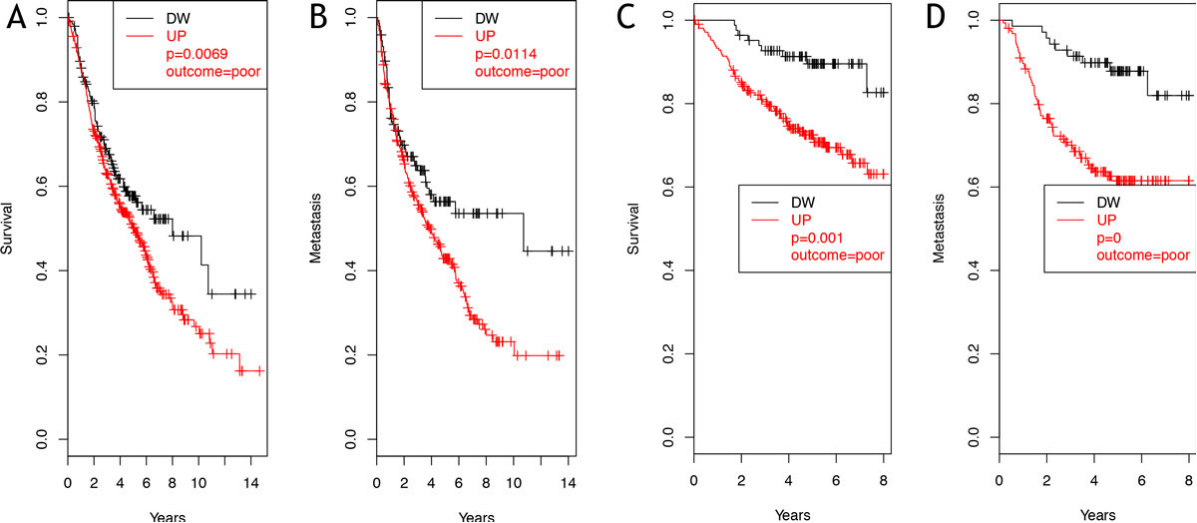
**SPP1 clinical outcome evaluation**. SPP1 showed a significant (p < 0.05) poor outcome in case of over-expression for both survival, in test (A) and validation data sets (C), and metastasis formation, in test (B) and validation data sets (D).

**Figure 4:**
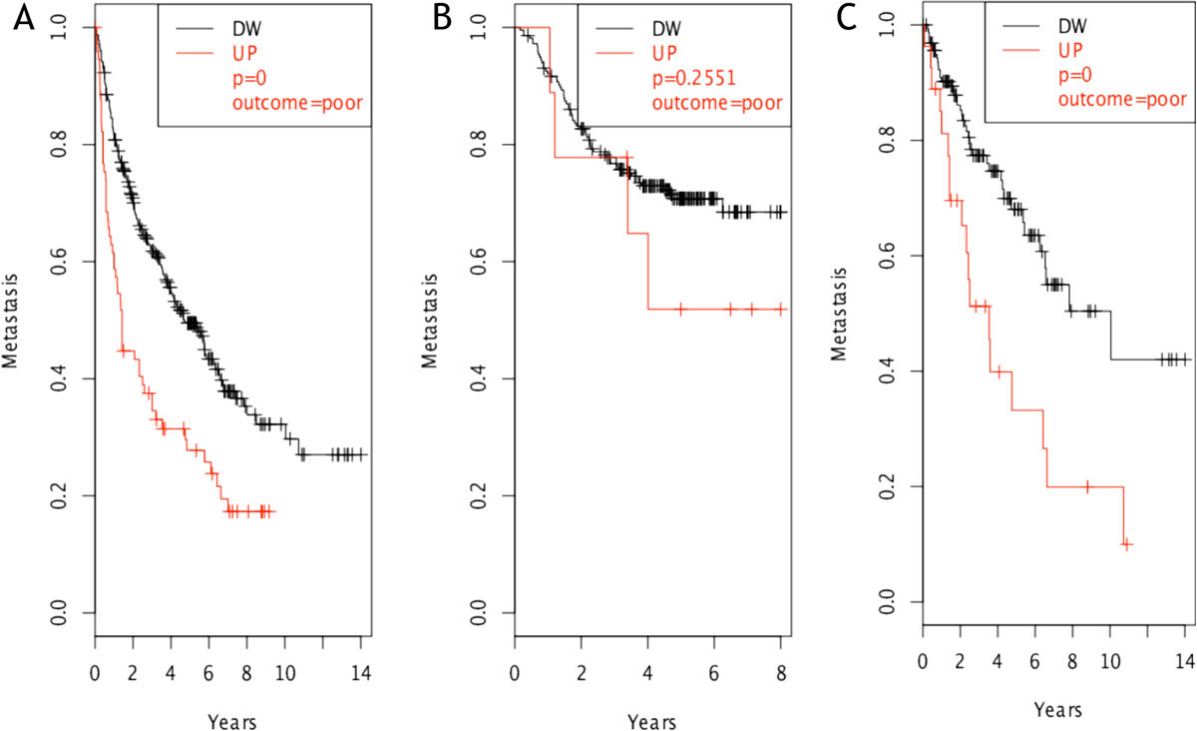
**GM-CSF clinical outcome evaluation**. GM-CSF showed a significant (p < 0.05) poor outcome regarding metastasis formation in case of over-expression in the test dataset (A). The significance was lost in the validation dataset (B), probably because of lack of sufficient data. Significance in test dataset was maintained when considering only early stage tumors (C).

**Figure 5:**
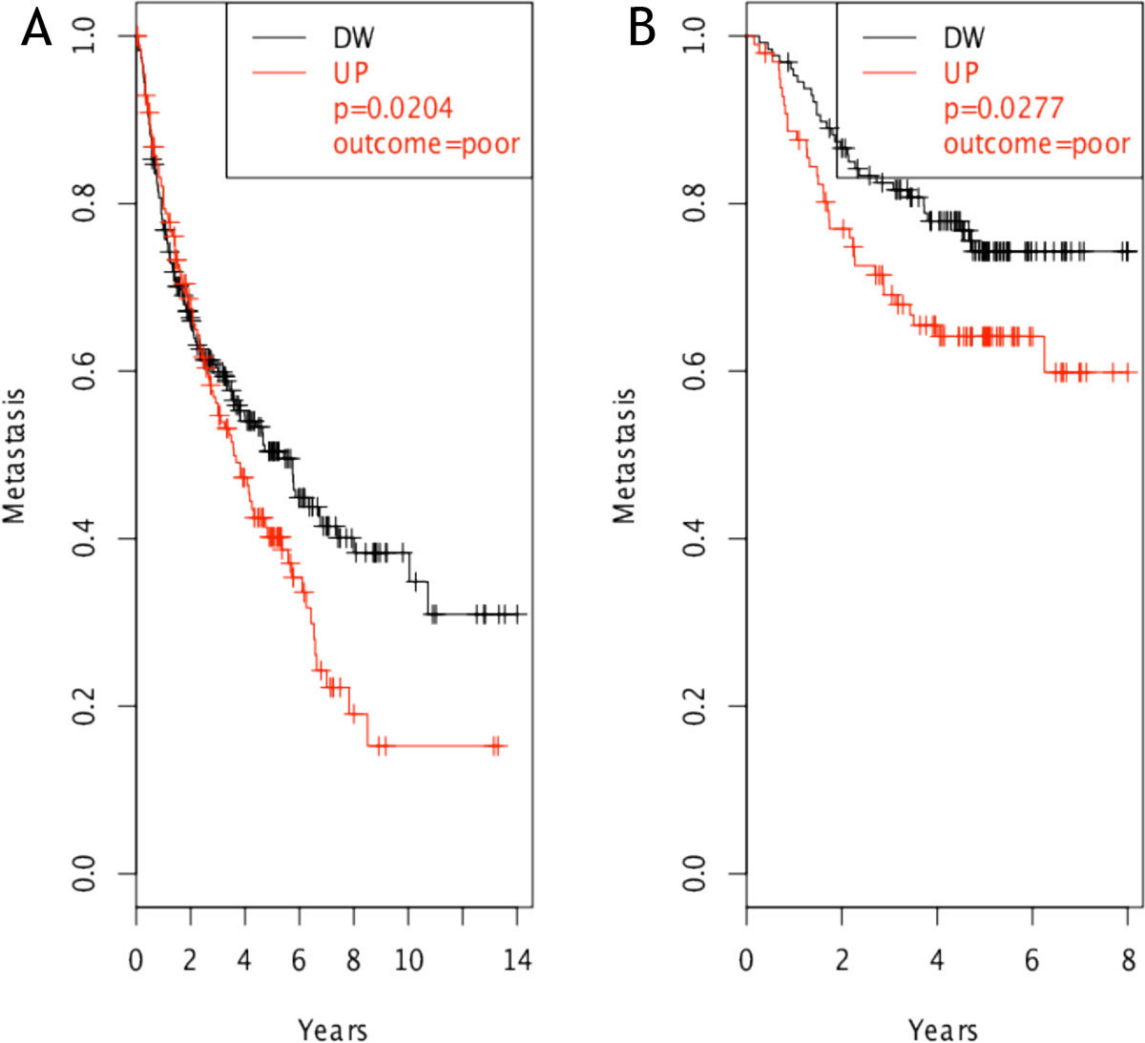
**ADORA3 clinical outcome evaluation**. ADORA3 showed a significant (p = 0.05) poor outcome regarding metastasis formation in case of over-expression in both test (A) and validation (B) datasets we considered. Its role is connected to late stages of cancer development (> 2 years).

SPP1 (osteopontin) was the only molecule whose over-expression resulted statistically related to poor outcome regarding both survival and metastasis formation in NSCLC patients examined (Figure 3). This result was further maintained in both datasets evaluating only early tumor stage samples, i.e. category T1 based on the TNM staging system [26]. These results are in accordance with previous evidences that SPP1 is an early marker of tumor progression in NSCLC [27,28]. Among the identified genes, two additional molecules showed a significant over-expression in patients with poor outcome regarding metastasis formation: GM-CSF (Figure 4) and ADORA3 (Figure 5).

**Figure 6:**
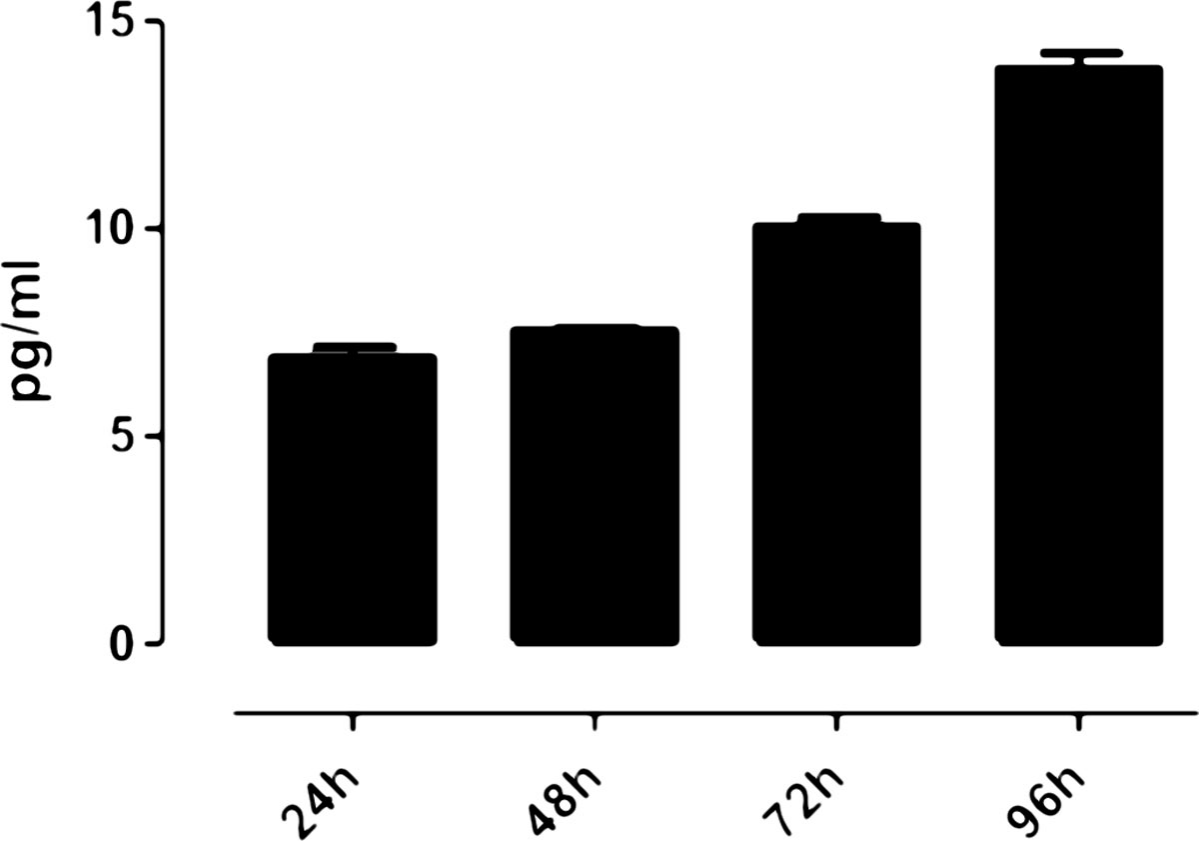
**GM-CSF production by KP cells**. The presence of GM-CSF was tested in the supernatant of KP cells after 24, 48, 72 and 96 hours of culture by ELISA. Results are expressed as the mean of three different supernatants ± SEM. The experiment was performed three times and a representative one is here shown.

GM-CSF, the granulocyte and macrophage colony stimulating factor, is a monomeric, 4-helical, secreted cytokine known to inhibit inflammation and T-cell immunity [29]. It has been described to promote cancer in pancreatic ductal neoplasia when over-expressed by a constitutively active form of K-ras [30], in accordance with our previously observed results in K-ras^LA1^/p53^R172HΔg ^mice. The association of GM-CSF expression with poor outcome was obtained in the test dataset. The result could not be confirmed in the validation dataset probably due to the limited number of samples in high expression cluster (Figure 4B, red curve). Nevertheless, significance in the first dataset was maintained even only considering early stage T1 tumors (Figure 4C). Analysis of the supernatants from a cell line (KP cells) derived from a lung tumor of a 30 week-old K-ras^LA1^/p53^R172HΔg ^mouse confirmed that they express GM-CSF (Figure 6). Taken together our data, with the observation that serum level of GM-CSF is significantly higher in colon adenocarcinoma patients [31], suggest that GM-CSF might represent a putative early marker in lung adenocarcinoma detection.

ADORA3 is a member of a family of 7-transmembrane G-protein-coupled receptor for adenosine. It has been reported to be involved in cell cycle regulation and tumor growth control both in vitro and in vivo [32]. It has been recently shown [33] that ADORA3 is involved in the induction of p53-mediated apoptosis in lung cancer cell lines. Since in our model p53 is inactivated, ADORA3 does not negatively affect tumor growth, but remain expressed on tumor cells. Although it does not represent a suitable oncoantigen, since its expression does not strictly affect tumor behavior; however, since it is a tumor associated antigen it could represent an interesting target for the development of antibody-mediated therapy on the subset of NSCLC which are p53 null and ADORA3 positive.

## Conclusions

The combination of powerful transcriptomics analysis, i.e. RNA-seq, genetically engineered mice models prone to develop tumors and large collection of human tumor transcriptomes offers new opportunities for the discovery and validation of therapeutic targets in the framework of personalized medicine. The identification of a known biomarker as osteopontin in the NSCLC mouse model confirmed the efficacy of our pipeline to detect targets in precision medicine. Moreover, our approach also allowed the identification of a new putative target, ADORA3, as well as a new putative biomarker, GM-CSF.

## Methods

### Mice

The heterozygous K-ras^LA1 ^mice were crossed with heterozygous p53^R172HΔg ^mice (both kindly provided by Dr. G. Lozano, University of Texas, Houston, TX, USA) to generate K-ras^LA1^/p53^R172HΔg ^and WT mice. The background of these mice was 129/Sv. Mice were maintained in the transgenic unit of the Molecular Biotechnology Center (University of Torino) under a 12 hour light-dark cycle and provided food and water ad libitum. Genotyped and individually tagged mice of the same age were treated in conformity with national and international laws and policies as approved by the Faculty Ethical Committee and all animal experiments were performed in accordance with European Union guidelines and national institutional regulations. Genotyping of K-ras^LA1 ^mice was performed as previously described [13]. To determine p53^R172HΔg ^mouse genotypes, PCR analysis was performed on tail DNA using the following primer sets: BMGFD (covering part of intron 4 and of the exon 5; 5'- TCT CTT CCA GTA CTC TCC TC -3') and BMGRV (covering the end of exon 7 and part of intron 7; 5'- GCC TTC CTA CCT GGA GTC TT -3') (Invitrogen Corp., Carlsbad, CA) for the amplification of p53 allele. The resulting PCR product was then digested with HgaI restriction enzyme (Invitrogen) to discriminate p53 WT from p53^R172HΔg ^mutant alleles.

### Cell line

KP is a cloned cell line established in vitro from a lung carcinoma that arose spontaneously in a K-ras^LA1^/p53^R172HΔg ^mouse. KP cells were cultured in DMEM with Glutamax 1 (DMEM, Life Technologies) supplemented with 20% heat-inactivated fetal bovine serum (Invitrogen).

### Magnetic Resonance Imaging (MRI)

MR images were acquired on a Bruker Avance 300 (Bruker, Ettlingen, Germany) operating at 7T using a 30 mm insert birdcage. Mice at different weeks of age (i.e. 10, 20 and 30 weeks, n = 3 each group) were anesthetized by injecting intramuscularly a mixture of tiletamine/zolazepam 20 mg/kg (Zoletil 100; Virbac, Milperra, Australia) and 5 mg/kg xylazine (Rompun; Bayer, Milano, Italy). Breath rate was monitored throughout in vivo MRI experiments using a respiratory air pillow (SA Instruments, Stony Brook, NY).

T_2_w axial, coronal and sagittal MR images with an in-plane resolution of 100 μm were acquired with a breath-triggered sequence respiratory gating to reduce lung movement artefacts using a RARE sequence (typical setting TR/TE/NEX/RARE factor = 6.0 s/4.14 ms/2/16) preceded by a fat-suppression module. A 256 × 256 acquisition matrix was used with a field of view of 25 × 25 mm^2^. The slice thickness was 1 mm, and the number of slices was 18 to 20, which was sufficient to cover the entire lung so that tumor volume could be measured. The T_2_w sequence can display the tumor location, size, and shape in both left and right lungs, providing clear boundaries with normal lung tissue.

### Tumor Volume Measurements

Data analysis of MR images was performed by using an open source application, ITK-Snap (http://www.itksnap.org), for segmentation of the lung nodules in three-dimensions, calculating both the number and the size of tumor lesions [34]. Tumor volume per animal was quantified by calculating the area of visible lung opacities hyper intense regions present in each axial or coronal image slice sequence (usually 18-20 per mouse) and then multiplying the sum of the areas by the distance between each MRI sequence slice. The post-processing of the segmented data provides the voxel counts and the volume (mm^3^) and displays the shape of the segmented structure. Tumor volumes were normalized relative to the total lung volumes at the indicated times and expressed as percentage of lung volume occupied by tumors.

### Lung tumor collection

Normal lung tissues and primary lung adenocarcinomas were collected from WT and K-ras^LA1^/p53^R172HΔg ^mice, at different stages of cancer progression (corresponding to 10, 20 and 30 weeks of age). Groups of three to six WT and K-ras^LA1^/p53^R172HΔg ^mice were sacrificed by cervical dislocation at the indicated times. Specimens for RNA extraction and gene expression profile analysis were stored in RNA later (Sigma-Aldrich, Milano, Italy) at 4° C for 24 h and then snap-frozen in liquid nitrogen and stored at -80° C until use. Tissues for histological and immunohistochemical studies were fixed in 10% neutral-buffered formalin and embedded in paraffin.

### Histopathological and immunohistochemical analysis

Tumors and tissues collected from K-ras^LA1^/p53^R172HΔg ^mice were fixed in formalin or PLP (Paraformaldehyde/Lysine/Periodate) and embedded in paraffin or frozen in OCT, respectively. Sections were stained with hematoxylin and eosin (H&E) for histological evaluation. Immunohistochemical staining was performed with the following primary antibodies: anti-TTF-1 (Thyroid Transcription Factor-1), anti-p63, anti-Synaptophysin and anti-Neuron Specific Enolase (NSE). Slides were then incubated with the appropriate biotinylated secondary antibody. Immunoreactive antigens were detected using NeutrAvidin™ Alkaline Phosphatase Conjugated (Thermo Scientific-Pierce Biotechnology, Rockford, USA) and Vulcan Fast Red (Biocare Medical, Concord, CA) or DAB Chromogen System (Dako Corporation, Carpinteria, CA, USA).

### RNA extraction

Total RNA was isolated from lung specimens by using an IKA-Ultra-Turrax^® ^T8 homogenizer (IKA-Werke, Staufen, Germany) and TRIzol^® ^reagent (Invitrogen), according to the manufacturer's instructions. Genomic DNA contaminations were removed from total RNA by using DNA-free kit (Ambion, Warrington, England) as per manufacturer's instructions. Total RNA concentration and purity were assessed using NanoVue Plus Spectrophotometer (GE Healthcare, Milano, Italy); RNA quality was evaluated on an Agilent 2100 Bioanalyzer following the manufacture's recommendations (Agilent Technologies, Milano, Italy), with a RNA integrity number (RIN) greater than 8.0 considered acceptable for expression profiling by microarray.

### Microarray data generation and analysis

Total RNA was then used to create the biotin-labelled cDNA probes to be hybridized on GeneChips Exon 1.0 ST mouse microarrays following the procedure described by the manufacturer (Affymetrix, Santa Clara, CA). Arrays were scanned on Affymetrix Gene ChIP Scanner 3000 7G and the CEL files were analysed as follows.

The CEL files resulting from the analysis of image files were analysed using oneChannelGUI 1.6.5 [35]. Gene-level expression was calculated using RMA method (Robust Multichip Average) [36] and normalized by quantile sketch method [37].

The gender effect was modelled to evaluate if any gene was associated to the difference in tumor growth observed between males and females.

The maSigPro Bioconductor library was used to assess differential expression at gene level [38]. maSigPro statistics follows a two-step regression strategy. It first adjusts the model by the least squared technique to identify differentially expressed genes and selects significant genes applying false discovery rate control procedures (FDR ≤ 0.05). Secondly, backward stepwise regression is applied to study differences between experimental groups (p ≤ 0.05). The final list of significant differentially expressed genes is defined using the R^2 ^values (R^2 ^≥ 0.6) of this second step.

Data were deposited on GEO database: GSE30878

### RNA-seq and transcriptome analysis

RNA libraries were sequenced using (HiSeq2000, Illumina, CA, USA). Two pools of total RNA extracted from 30 week-old mice (n = 3) were generated for WT and MT. Each pool was sequences twice to increase the coverage. A total of 51,756,477 and 70,406,984 paired-end (PE) reads were obtained for the first and the second MT replicates, respectively. In the case of the WT replicates 79,079,459 and 63,675,355 PE reads were observed, respectively. Data were deposited on GEO database: GSE51144

#### Fusion detection

De-novo discovery of chimeric transcripts was done by ChimeraScan [22] with default parameters. For the first and the second MT datasets 5066 and 4543 putative events were measured, respectively. 4533 and 4351 putative events were found for the first and second WT dataset, respectively. Gene fusions were annotated using chimera Bioconductor package. Only the fusion events in common between replicates were retained.

#### Gene/Exon-level analysis

Reads were mapped on mouse reference genome mm9 using TopHat version 2.0.4, using default parameters and UCSC annotation (http://genome.ucsc.edu/).

Mapped reads were counted for each replicate of WT and MT using DEXSeq package [24]. Briefly, dexseq_count.py script was used to associate reads to exons and differentially expressed exons were detected using FDR < 0.1 and |log_2_Fold Change| > 1.

Then, geneCountTable function was used to collapse exon-level in gene-level counts. Differential expression was subsequently evaluated using DESeq package [25] (FDR < 0.1, |log_2_Fold Change| > 1).

### Collection and processing of lung cancer expression data

#### Microarrays

Seven datasets containing microarray data of lung cancer samples (adenocarcinoma and squamous cell carcinoma) and annotations on patients' clinical outcome were collected. All data were measured on different Affymetrix arrays and have been downloaded from NCBI Gene Expression Omnibus (GEO, http://www.ncbi.nlm.nih.gov/geo/), caArray (https://array.nci.nih.gov/caarray/home.action), and the Computational Biology Center of the Memorial Sloan-Kettering Cancer Center (http://cbio.mskcc.org/). The complete list of datasets is provided in Table 1.

**Table 1:** Original lung cancer datasets

Source	Affymetrix platform	Samples	References
GEO GSE3141	HG-U133 Plus 2.0	111	Bild et al., 2006 [51]
GEO GSE19188	HG-U133 Plus 2.0	156	Hou J et al., 2010 [40]
caArray jacob-00182	HG-U133A	468	Shedden et al., 2008 [52]
http://cbio.mskcc.org/Public/lung_array_data/	HG-U133Av2	129	Nguyen et al., 2009 [42]; Chitale et al., 2009 [43]
GEO GSE10245	HG-U133 Plus 2.0	58	Kuner et al., 2009 [44]
GEO GSE31210	HG-U133 Plus 2.0	226	Okayama H et al., 2012 [45,53]
GEO GSE14814	HG-U133A	90	Zhu CQ et al., 2010 [54]

Prior to analysis, the datasets were reorganized by eliminating duplicate samples and samples without outcome information. Briefly, the original studies have been modified as follows: GSE3141 [39] has been re-named as Duke (Duke University) and used as it is; GSE19188 [40] has been re-named EMC and used after removal of samples lacking the patient outcome information; Shedden [41] has been split into MI (187 samples from the University of Michigan Cancer Center), DFCI (82 samples of the Dana-Farber Cancer Institute); HLM (92 samples collected at the Moffitt Cancer Center), and MSKCC_1 (107 samples from the Memorial Sloan-Kettering Cancer Center); Ladanyi-Gerald [42,43] has been re-named as MSKCC_2 (Memorial Sloan-Kettering Cancer Center) and used as it is; GSE10245 [44] has been re-named DKFZ (German Cancer Research Center) and used as it is; GSE31210 [45] re-named NCCRI (National Cancer Center Research Institute, Japan) and used as it is; GSE14814 [45] re-named OCI-PMH (Ontario Cancer Institute, Princess Margaret Hospital) and used after removal of large cell undifferentiated carcinoma samples. This re-organization resulted in a compendium (meta-dataset) comprising 989 unique adenocarcinoma samples from seven independent cohorts. The type and content of clinical and pathological annotations of the meta-dataset samples have been derived from the original cohorts.

According to Cordenonsi et al., [46] clinical information among the various datasets was standardized redefining the outcome descriptions based on the clinical annotations of each individual study. Specifically, we defined two major types of events, i.e., metastasis and survival.

Raw expression data (i.e., CEL files) obtained from different platforms was integrated using an approach inspired by geometry and probe content of HG-U133 Affymetrix arrays [47]. Briefly, probes with the same oligonucleotide sequence, but located at different coordinates on different type of arrays, have been arranged in a virtual platform grid. As for any other microarray geometry, this virtual grid has been used as a reference to create a virtual Chip Definition File (virtual-CDF), containing probes shared among the various HG-U133 platforms and their coordinates on the virtual platform, and a virtual-CEL file containing the fluorescence intensities of the original CEL files properly re-mapped on the virtual grid. Expression values for 21981 meta-probesets were generated from the transformed virtual-CEL files using a virtual-CDF obtained merging HG-U133A, HG-U133Av2, and HG-U133 Plus2 original CDFs. Fluorescence signals were background adjusted, normalized using quantile normalization, and gene expression levels calculated using median polish summarization (RMA; [48]). The entire procedure was implemented as an R script. The meta-dataset is available upon request to the authors.

#### RNAseq

Public RNA sequencing human lung adenocarcinoma data and related clinical metadata were downloaded from The Cancer Genome Atlas repositories (http://cancergenome.nih.gov/). Two datasets were available at the day of the download, containing respectively a total of 162 (RNASeq) and 452 (RNASeqV2) samples with exon-level expressions. After filtering the transcriptomes on the basis of the available clinical annotations we obtained a dataset of 509 NSCLC adenocarcinoma transcriptomes (Additional file 1). The entire procedure was implemented as an R script.

### Clinical Outcome score evaluation

The microarray meta-dataset was split in two separate groups containing respectively 695 (from cohorts published between 2005 and 2009) and 294 samples (from cohorts published between 2011 and 2012).

Exon-level analysis was done on 137 and 372 samples derived from Cancer Genome Atlas RNASeq dataset and from RNASeqV2 dataset, respectively

Expression levels of each putative target (gene/exon) discovered by the analysis of RNA-seq data were divided in two clusters using a k-means clustering (k = 2). Median expression for each cluster was calculated. The label "UP" was associated to the cluster characterized by the higher median expression, while the other cluster was labelled "DOWN".

Exponential survival models [49] from the survival R package, were fitted for the UP and DOWN clusters and the significance (P_true_) of the differences between the models were tested [50]. Then, we performed a random assignment of UP and DOWN labels to the samples and we tested the significance (P*) of the difference between these null models. This procedure was repeated n times (n = 10000), randomly removing, at each repetition step, 10% of the samples.

Clinical Outcome score (CO) was then calculated with the following formula:

CO=on

FR and EQ generated the animal model and prepared samples for histological and microarray analysis, MA and AF prepared samples for RNA-seq, GB and EZ sequenced the RNA-seq libraries, MI did the histological analyses, DLL run the NMR analyses. MC and RAC did transcriptome data analysis; SN and SB prepared the lung transcriptome dataset. PN and LL generate the KP cell line. RAC, FC and EQ conceived, designed and supervised the study, and wrote the paper.

## Competing interests

The authors declare that they have no competing interests.

## Declaration

The publication costs for this article were funded by grants from the Italian Association for Cancer Research; the Epigenomics Flagship Project EPIGEN.

This article has been published as part of BMC Bioinformatics Volume XX Supplement X, 2014: Italian Society of Bioinformatics (BITS): Annual Meeting 2013.

This article has been published as part of *BMC Genomics *Volume 15 Supplement 5, 2014: Italian Society of Bioinformatics (BITS): Annual Meeting 2013: Genomics. The full contents of the supplement are available online at http://www.biomedcentral.com/bmcgenomics/supplements/15/S3
